# Disability and Biomedical HIV Prevention: The Missing Equity Dimension

**DOI:** 10.3390/v18091017

**Published:** 2026-09-14

**Authors:** Francesco De Maria, Paolo Fusco, Alessandro Russo

**Affiliations:** Infectious and Tropical Diseases Unit, Department of Medical and Surgical Sciences, “Magna Graecia” University of Catanzaro, 88100 Catanzaro, Italy; francescodemaria16@gmail.com (F.D.M.);

**Keywords:** HIV prevention, pre-exposure prophylaxis (PrEP), Disability, health equity, implementation science

## Abstract

The remarkable success of biomedical HIV prevention has transformed the global response to HIV through the widespread implementation of oral pre-exposure prophylaxis (PrEP), long-acting antiretroviral agents and HIV self-testing. However, ensuring equitable access to these interventions remains a major challenge. Although more than one billion people worldwide live with a disability, disability has received surprisingly little attention within contemporary HIV prevention research and implementation. This narrative review examines the intersection between disability and modern HIV prevention, integrating evidence on sexual and reproductive health, structural barriers to healthcare and the evolving landscape of biomedical prevention. The available literature suggests that people with disabilities experience persistent inequalities in access to sexuality education, HIV information, testing and preventive services, largely due to physical, communication and systemic barriers rather than biological vulnerability. Despite these well-documented disparities, disability is rarely incorporated into PrEP implementation studies, HIV prevention policies or differentiated service delivery models. We argue that disability should be considered an implementation and health equity challenge rather than a pharmacological one. The effectiveness of biomedical prevention ultimately depends on healthcare systems capable of delivering accessible, person-centred and inclusive services. Integrating disability into implementation science, clinical practice and public health policy represents an essential step toward achieving equitable HIV prevention. As HIV prevention continues to evolve, accessibility should be recognised not as an optional adaptation but as a fundamental component of high-quality prevention programmes.

## 1. Introduction

Over the past decade, HIV prevention has undergone one of the most profound transformations in the history of infectious diseases. Once dominated by behavioural interventions—including condom promotion, risk-reduction counselling and partner notification—it has progressively evolved into a model in which biomedical strategies play a central role. Oral tenofovir-based pre-exposure prophylaxis (PrEP), long-acting injectable cabotegravir and, in selected settings, the dapivirine vaginal ring, together with HIV self-testing and integrated sexual health services, have reshaped prevention programmes worldwide, demonstrating that HIV acquisition can be substantially reduced when effective interventions are delivered before exposure rather than after infection [1].

This evolution has also changed the way prevention is conceptualised. HIV prevention is no longer limited to modifying individual behaviour but increasingly depends on healthcare systems capable of identifying people at risk, ensuring appropriate clinical follow-up, supporting adherence and delivering prevention in an equitable and person-centred manner. Consequently, accessibility has become as important as efficacy. Even highly effective biomedical interventions cannot achieve their population-level impact if individuals are unable to access, initiate or remain engaged in prevention programmes.

Against this background, disability has received remarkably little attention. More than one billion people worldwide—approximately one in six of the global population—live with a disability, making disability a major dimension of global health equity recognised by the World Health Organization [2]. Yet disability has rarely occupied a prominent place within HIV prevention research, despite growing recognition that people with disabilities experience substantial disparities across multiple areas of healthcare delivery [3,4].

Historically, this neglect has been influenced, at least in part, by persistent assumptions that people with disabilities are sexually inactive or at inherently low risk of HIV acquisition. Such assumptions have contributed to the limited inclusion of people with disabilities in sexuality education, HIV prevention initiatives, epidemiological research and implementation studies [3,4,5]. This relative invisibility may itself perpetuate an evidence gap: limited research reduces the visibility of disability within policy and programme design, while limited inclusion in prevention programmes constrains opportunities to generate disability-specific evidence.

The literature accumulated over the past two decades challenges these assumptions. People with disabilities establish intimate relationships, are sexually active and may be exposed to many of the same determinants of HIV acquisition as people without disabilities [6,7]. In several settings, additional social and structural vulnerabilities have been documented, including lower access to education, poverty, dependence on caregivers, gender-based inequalities, social exclusion and increased exposure to sexual violence [3,4,8,9]. These conditions should not be interpreted as establishing a uniformly increased biological or behavioural risk of HIV across all people with disabilities. Rather, they may increase vulnerability in particular populations and settings while simultaneously limiting access to prevention [10].

Evidence also documents persistent barriers to sexual and reproductive healthcare. Across diverse healthcare settings, people with physical, sensory, intellectual and developmental disabilities have reported difficulties accessing sexual health information, HIV testing, contraception, counselling and preventive services [7,11,12,13,14,15,16]. The nature and magnitude of these barriers vary according to disability type and context. Physical inaccessibility may be particularly relevant for people with mobility limitations, communication barriers for people with sensory disabilities, and inaccessible information or consent processes for some people with intellectual or cognitive disabilities. At the same time, inadequate professional training, stigma, discriminatory attitudes and poorly adapted healthcare environments can affect people across disability groups [12,13,14,15,16,17,18,19]. These findings emphasise that many barriers arise from the interaction between individual accessibility needs and the organisation of healthcare services, rather than from disability alone [2,15].

Paradoxically, while research on disability and sexual health has expanded considerably, contemporary biomedical HIV prevention has developed with relatively limited explicit consideration of disability within its research and implementation frameworks. Current discussions surrounding oral and long-acting PrEP and HIV self-testing increasingly emphasise equity, implementation science and differentiated service delivery, yet disability remains comparatively underrepresented in these discussions. This disconnect is important because the successful delivery of biomedical prevention depends on healthcare processes—including accessibility, communication, continuity of care and health literacy—that may present specific challenges for different groups of people with disabilities [11,13,14,15,20,21,22].

Importantly, direct evidence evaluating the efficacy, uptake and implementation of contemporary PrEP modalities specifically among people with disabilities remains limited. Disability itself does not provide a basis for assuming reduced pharmacological efficacy; however, disability-specific pharmacokinetic, pharmacodynamic and clinical endpoint data are sparse, and different prevention modalities may present distinct accessibility advantages and disadvantages depending on disability type and care context. The key question is therefore not simply whether biomedical prevention can work in people with disabilities, but whether prevention systems are designed to ensure equitable access, informed choice, initiation and sustained use. As HIV prevention becomes increasingly dependent on engagement with healthcare services, disability should therefore be considered an important dimension of equitable implementation rather than treated as a homogeneous marker of biological vulnerability [21].

In this narrative review, we examine the intersection between disability and contemporary HIV prevention by bringing together evidence that has largely evolved in parallel. We first summarise current knowledge regarding disability, sexual and reproductive health, and HIV vulnerability before examining how structural and healthcare-related barriers may influence access to biomedical prevention. We then consider how these barriers and accessibility needs may differ across disability types and prevention modalities. Finally, we propose a framework for disability-inclusive HIV prevention that places accessibility, equity and implementation science at the centre of future research, policy development and clinical practice.

## 2. Narrative Review Approach

This narrative review was guided by the following research question: In the PrEP era, how are people with disabilities represented in HIV prevention research, guidelines, and implementation models? A structured narrative literature search was conducted in PubMed between 10 June and 10 July 2026. An updated guideline search was conducted in September 2026 to identify relevant guidance published after completion of the original literature search. The search was designed to capture both direct evidence addressing PrEP and disability and the broader literature relevant to potential barriers across the HIV prevention continuum.

The core PubMed search was: ((“pre-exposure prophylaxis”[Title/Abstract] OR PrEP[Title/Abstract]) AND (HIV[Title/Abstract] OR “human immunodeficiency virus”[Title/Abstract]) AND (disabilit*[Title/Abstract] OR disabled[Title/Abstract] OR “people with disabilities”[Title/Abstract])). Ten complementary thematic searches were conducted by combining disability-related terms with terms addressing PrEP access and implementation, broader HIV prevention, sexual and reproductive health services, healthcare access barriers, sexuality and HIV education, sexual violence and vulnerability, key populations, physical and mobility disabilities, sensory, intellectual and cognitive disabilities, and existing narrative, systematic and scoping reviews.

The PubMed searches identified 197 records, which were assessed for relevance to the objectives of this narrative review. Studies were considered for inclusion if they addressed people with disabilities in relation to HIV prevention, HIV testing, sexual and reproductive health, healthcare accessibility, or structural and implementation barriers potentially relevant to biomedical HIV prevention. Only English-language publications were considered. Studies without substantive relevance to disability or HIV and sexual health prevention, as well as records in which “PrEP” referred to concepts other than HIV pre-exposure prophylaxis, were excluded. Initial literature selection was performed by all three authors (F.D.M., P.F., and A.R.), with uncertain or borderline records discussed jointly and resolved by consensus. Because this was designed as a narrative rather than a systematic review, formal independent duplicate screening and statistical assessment of inter-reviewer agreement were not prespecified.

Current international HIV prevention guidelines issued by the World Health Organization (WHO), Centers for Disease Control and Prevention (CDC), European AIDS Clinical Society (EACS), British HIV Association (BHIVA), and International Antiviral Society–USA (IAS–USA) were additionally examined to assess the extent to which disability and accessibility were explicitly incorporated into contemporary HIV prevention recommendations. Guideline documents were searched for the terms disability, disabled, accessibility, impairment, wheelchair, cognitive, intellectual, deaf, hearing, blind, visual, and reasonable accommodation. Relevant passages were reviewed in context rather than considering term occurrence alone. Reference lists of relevant publications were also manually screened to identify additional studies.

**Figure 1:**
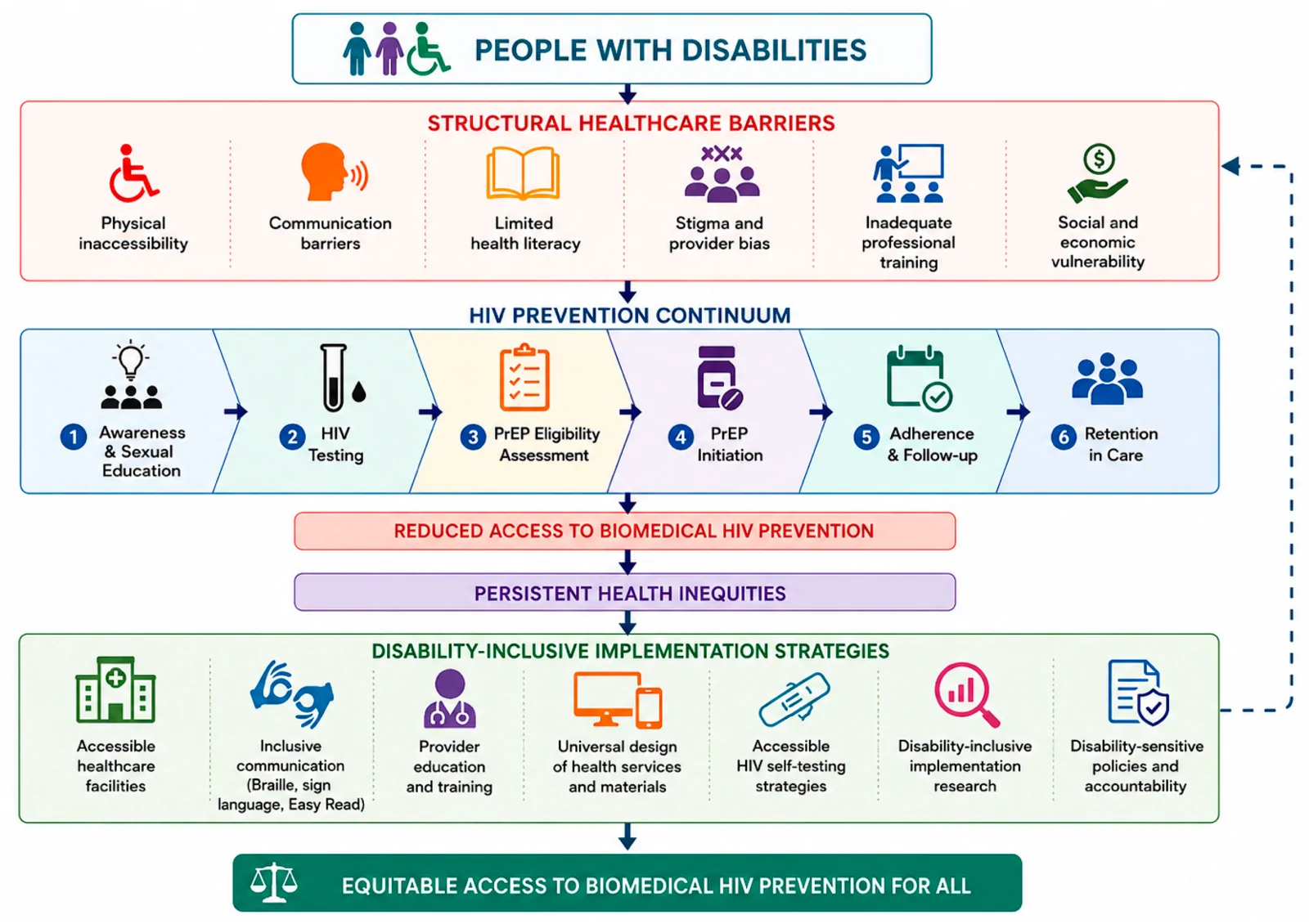
The Accessibility Continuum of Biomedical HIV Prevention: A Disability-Inclusive Framework. Structural barriers associated with disability—including physical inaccessibility, communication barriers, limited health literacy, stigma, inadequate professional training and social vulnerability—may affect every stage of the HIV prevention continuum, from sexual health education and HIV testing to PrEP eligibility assessment, PrEP initiation, adherence and long-term retention in care. These barriers can reduce equitable access to contemporary biomedical HIV prevention despite the proven effectiveness of available interventions. Disability-inclusive implementation strategies, including accessible healthcare environments, inclusive communication, provider education, universal design principles and disability-sensitive policies, represent potential approaches to support more equitable access to HIV prevention. The figure was generated using ChatGPT image generation (OpenAI, San Francisco, CA, USA; https://chatgpt.com/, accessed on 10 September 2026) and subsequently manually reviewed by the authors for conceptual accuracy and consistency with the reviewed literature.

Given the heterogeneous and relatively limited literature directly addressing biomedical HIV prevention among people with disabilities, evidence from the broader disability, sexual and reproductive health, and healthcare accessibility literature was also considered when relevant to the implementation of HIV prevention. Priority was given to systematic reviews, meta-analyses, international guidelines, recent empirical studies, and original studies directly relevant to the objectives of this review. Overall, 49 sources contributed to the final narrative synthesis, including peer-reviewed publications, international guidelines, and institutional reports. The literature search was intended to provide a structured and transparent evidence base for narrative synthesis rather than an exhaustive systematic evidence inventory. Accordingly, no quantitative synthesis or formal risk-of-bias assessment was undertaken. Figure 1 was generated using ChatGPT image generation (OpenAI, San Francisco, CA, USA; https://chatgpt.com/, accessed on 10 September 2026) and subsequently manually reviewed by the authors for conceptual accuracy, consistency with the reviewed literature, and alignment with the framework presented in the manuscript.

## 3. Disability and HIV: From an Overlooked Population to an Overlooked Equity Dimension

For much of the HIV epidemic, disability occupied a peripheral position within both research and public health policy. This marginalisation did not arise because evidence demonstrated a lower risk of HIV among people with disabilities; rather, it reflected, at least in part, a long-standing assumption that disability was incompatible with sexuality. The widespread perception that people with disabilities were sexually inactive or substantially less likely to engage in sexual relationships contributed to their limited visibility within prevention programmes, surveillance systems and clinical practice [5,7]. As a result, disability remained largely overlooked within the global HIV response [3,4].

The consequences of this misconception are important. When a population is perceived as being at negligible risk, its prevention needs may receive less attention in epidemiological surveillance, prevention campaigns and implementation research. Disability therefore became a blind spot within HIV prevention—not because evidence established its irrelevance, but because disability-specific questions were rarely incorporated into research and programme design [3,4].

Evidence accumulated over the last twenty years has challenged this narrative. People with disabilities are sexually active, establish long-term relationships, marry, have children and experience many of the same sexual health needs as individuals without disabilities [6,7]. Available studies indicate that disability should not be regarded as inherently protective against HIV acquisition. Several population-based investigations and reviews have reported HIV prevalence comparable to, and in some settings higher than, that observed among people without disabilities [10,23,24,25]. However, estimates vary substantially according to geographical setting, population characteristics and disability type, and the available evidence does not support the assumption of a uniform increase in HIV risk across all people with disabilities. Rather, it demonstrates that disability cannot be equated with protection from HIV and that specific groups may experience greater vulnerability in particular social and healthcare contexts.

Understanding these patterns requires moving beyond biological explanations. Disability does not inherently increase biological susceptibility to HIV infection. Rather, it may intersect with social and structural determinants that influence both exposure to HIV and access to prevention. Poverty, unemployment, lower educational attainment, gender inequality, dependence on caregivers and social exclusion have been documented among different populations of people with disabilities and may coexist within the same individual [2,4,7]. Their distribution and impact are not uniform across disability groups or settings, but they can create circumstances in which access to HIV information and prevention becomes more difficult [16]. This perspective is consistent with the contemporary understanding of disability proposed by the World Health Organization, which emphasises the interaction between health conditions and environmental or social barriers rather than disability as an individual deficit [2]. Applied to HIV prevention, this framework shifts attention from disability alone towards the interaction between individual accessibility needs and the systems responsible for delivering prevention.

Another recurring finding across the literature is increased exposure to sexual violence and coercion among some populations of people with disabilities. Meta-analyses have documented higher rates of sexual violence or abuse among children and adults with disabilities, with particularly important concerns for women and people with intellectual disabilities [9,26,27]. These experiences may be shaped by multiple and intersecting factors, including dependence on caregivers, communication barriers, institutionalisation, reduced autonomy, gender inequality and unequal power relationships. Sexual violence is relevant to HIV prevention because it may increase exposure to sexual transmission while also creating barriers to subsequent healthcare seeking and prevention. However, the available disability-specific literature more clearly establishes the increased burden of sexual violence than a direct causal pathway from disability-associated sexual violence to HIV acquisition. This distinction should be recognised when interpreting the available evidence.

Knowledge inequalities represent another persistent concern. Compared with peers without disabilities, adolescents and young adults with disabilities have been reported in several settings to have lower levels of HIV knowledge and reduced access to comprehensive sexuality education [11,28,29,30]. The nature of these barriers differs across disability types, and some people may require information and communication approaches adapted to sensory, intellectual or cognitive accessibility needs [19,31]. Discussions about sexuality may also be discouraged by families or professionals, while healthcare providers may underestimate the need for sexual health counselling among people with disabilities [6,15,17,18].

Despite increasing recognition of these disparities, disability remains insufficiently represented within some national HIV strategies and surveillance systems. Reviews of HIV strategic plans in sub-Saharan Africa have shown that disability may be absent or only superficially addressed within national responses, despite evidence documenting inequalities in HIV prevention and care [3,5,21,32]. More broadly, disability status is not routinely reported as a participant characteristic in much of the HIV prevention literature, limiting the ability to determine whether contemporary prevention programmes reach people with different types of disabilities equitably [21,22]. The available evidence is also geographically uneven, with substantially more information from sub-Saharan Africa and selected high-income settings than from several other world regions [3,4,7,21].

Taken together, these observations suggest that disability should not be approached simply as a homogeneous vulnerable population within HIV prevention. Rather, it can be understood as an equity dimension that intersects with gender, socioeconomic position, geography, sexual orientation, gender identity and other determinants. The specific barriers encountered may differ substantially according to disability type and individual context. Recognising disability in this way shifts the focus from assigning risk on the basis of disability status to asking whether contemporary prevention systems are sufficiently accessible and adaptable to provide equitable access to HIV prevention.

## 4. Sexual and Reproductive Health: The Foundation of Effective HIV Prevention

Although HIV prevention has traditionally been discussed within infectious diseases, its implementation is closely connected to the broader landscape of sexual and reproductive healthcare. Access to contraception, comprehensive sexuality education, counselling, STI screening and reproductive services forms part of the infrastructure upon which contemporary HIV prevention is built. Consequently, inequalities affecting sexual and reproductive healthcare may also influence access to HIV prevention, particularly as biomedical strategies become increasingly integrated within routine sexual health services.

For people with disabilities, substantial inequalities in sexual and reproductive healthcare have been documented. Over the past decade, systematic reviews conducted across diverse geographical settings have identified unmet sexual and reproductive health needs among people with physical, sensory, intellectual and developmental disabilities [6,12,13,14,17,18,20,33,34,35]. These disparities should not be interpreted as reflecting lower demand for care. Rather, the available literature describes barriers within healthcare and educational systems that may limit recognition of, and responses to, the sexual and reproductive health needs of people with disabilities.

One persistent concern involves assumptions surrounding sexuality. Despite growing societal awareness, people with disabilities may still be viewed predominantly through a medical or caregiving lens, with their sexuality overlooked or insufficiently addressed. Such attitudes can influence families, educational institutions and healthcare professionals, contributing to situations in which discussions regarding intimate relationships, contraception, sexually transmitted infections and HIV prevention are postponed, avoided or inadequately adapted to individual needs [6,16,18,30].

Educational inequalities may begin early. Adolescents with disabilities have been reported to receive less comprehensive sexuality education than their peers without disabilities, while the information available to them may be incomplete, inaccessible or poorly adapted to their communication needs [28,29,30,36]. Educational resources may not always be provided in formats accessible to people with visual or hearing impairments, while conventional written materials may not be appropriately adapted for some people with intellectual or cognitive disabilities [19,31]. Discussions surrounding consent, healthy relationships and HIV prevention may also be omitted when educators assume these topics are irrelevant [37].

Clinical encounters can reinforce these barriers. Studies conducted in both high-income and low- and middle-income settings describe physical inaccessibility, insufficient time to address communication needs and healthcare professionals who report limited preparation for discussing sexuality in the context of disability [12,15,17,18,20,38]. In some settings, conversations may be directed towards caregivers rather than the individual, potentially undermining autonomy and opportunities for supported or shared decision-making. In others, sexual health may not be discussed because of assumptions regarding sexual activity.

These barriers are particularly relevant because modern sexual healthcare often relies on longitudinal relationships between individuals and healthcare providers. Prevention may involve repeated engagement with healthcare systems rather than isolated clinical encounters. Whether the objective is contraception, cervical cancer screening, HIV testing or PrEP initiation, individuals need to be able to access services, communicate effectively with healthcare professionals and return for appropriate follow-up. For some people with disabilities, barriers at several points along this pathway may accumulate and ultimately influence access to prevention [16].

The literature also highlights the importance of recognising diversity within disability itself. The challenges experienced by a person with a mobility impairment may differ substantially from those encountered by a Deaf individual, a person with visual impairment or someone with an intellectual or cognitive disability. Physical accessibility may be particularly relevant for people with mobility limitations, whereas communication accessibility may be more prominent for people with sensory disabilities. Accessible information, supported decision-making and appropriately adapted counselling may assume greater importance for some people with intellectual or cognitive disabilities [12,15,16,17,18,19,31,38,39,40]. These categories are themselves heterogeneous and may overlap within the same individual. Treating disability as a single homogeneous category therefore risks obscuring distinct accessibility requirements.

Sexual violence represents an additional consideration within sexual and reproductive healthcare. Multiple systematic reviews and meta-analyses have documented higher rates of sexual violence among some populations of people with disabilities, with particularly important concerns for women and people with intellectual disabilities [9,26,27]. As discussed above, the evidence more clearly establishes this increased burden than a direct disability-specific causal pathway to HIV acquisition. Nevertheless, previous experiences of violence or coercion may affect healthcare seeking, trust and engagement with preventive services.

Taken together, the available evidence suggests that an important challenge lies not in an assumed lack of interest in sexual and reproductive healthcare among people with disabilities, but in the ability of healthcare systems to recognise and accommodate heterogeneous accessibility needs. This distinction is particularly relevant because contemporary HIV prevention increasingly operates within services where many of these barriers have already been documented. If sexual health services are difficult to access or navigate, the delivery of newer biomedical interventions may reproduce rather than overcome existing inequalities.

For this reason, disability should not be viewed solely through the lens of HIV prevention. It can also serve as an important indicator of whether sexual and reproductive healthcare systems are capable of delivering genuinely accessible and person-centred care. Strengthening this foundation will be important if biomedical HIV prevention is to achieve equitable population-level impact, including among people who have historically received limited attention within sexual health policy and practice [21].

## 5. Barriers Across the HIV Prevention Continuum: Where Inequities Emerge

The success of contemporary HIV prevention depends on far more than the availability of effective biomedical interventions. Unlike many traditional public health measures, prevention strategies such as PrEP, HIV self-testing and long-acting injectable prophylaxis are delivered through healthcare pathways that require sustained interaction between individuals and health systems. Awareness, access, eligibility assessment, counselling, HIV testing, PrEP initiation, adherence support and long-term follow-up are all necessary components of the prevention continuum. Failure at any one of these stages may compromise the overall effectiveness of prevention, regardless of the efficacy of the intervention itself. As illustrated in Figure 1, barriers documented in the broader disability and sexual healthcare literature may plausibly operate at multiple stages of the HIV prevention continuum, although direct empirical evidence linking specific disability-related barriers to PrEP outcomes remains limited [3,12,13,14,15,16,20].

From this perspective, disability represents an important dimension of healthcare accessibility that may influence different steps along the prevention pathway, depending on disability type, individual needs and service context [2,4]. The relevant question is therefore not simply whether people with disabilities may be exposed to HIV, but whether current prevention systems are sufficiently accessible and adaptable to ensure equitable access to effective interventions.

The first barrier often arises before individuals interact with healthcare services. Awareness of HIV prevention strategies depends heavily on comprehensive sexuality education, public health campaigns and community engagement. Yet available studies indicate that some groups of people with disabilities experience lower access to sexual health information and accessible educational resources [11,28,29,30,36,38]. Educational programmes and health promotion materials may not be adapted to sensory, cognitive or communication needs, while prevention campaigns may fail to explicitly consider disability alongside other dimensions of health equity. As a consequence, limited access to accessible information may reduce opportunities to learn about biomedical HIV prevention options before individuals encounter specialised prevention services [12,19,28,29,30,31,36,38].

Healthcare access represents the next critical step. Reaching an HIV or sexual health clinic may itself constitute a substantial challenge for some people with mobility or physical disabilities because of inaccessible buildings, limited transportation, architectural barriers or inadequate clinical equipment [3,12,13,15,33,34]. These obstacles are not unique to HIV prevention, but may become particularly relevant when prevention involves repeated clinical attendance and monitoring. PrEP delivery requires continuity of care, although the frequency and nature of healthcare contact differ according to the prevention modality used.

Communication represents another critical step in the continuum because HIV prevention relies on informed decision-making, shared understanding of HIV risk, medication counselling and adherence support. People with sensory disabilities may encounter information that is not provided in accessible formats, while individuals with intellectual disabilities may encounter communication that is either overly simplified or unnecessarily complex [17,18,19,31,37,38,40]. In some healthcare settings, discussions regarding sexual health may be directed towards caregivers rather than the individuals themselves, potentially limiting autonomy and opportunities for appropriately supported informed decision-making [17,18,40].

Provider attitudes and clinical practices may create additional barriers. Healthcare professionals may underestimate the sexual activity of people with disabilities or feel uncomfortable discussing sexuality [15,16,17,18,40]. Where sexual behaviours and prevention needs are not explored, opportunities to offer HIV testing or discuss PrEP may be missed. Direct evidence quantifying the contribution of provider attitudes to PrEP exclusion among people with disabilities remains limited, however, and these factors should be understood alongside broader structural, organisational and accessibility barriers.

The organisation of healthcare services may further amplify these disparities. HIV prevention has increasingly adopted differentiated service delivery models intended to improve convenience and retention in care. Nevertheless, the extent to which disability-specific accessibility requirements have been incorporated into these models remains poorly documented [21]. Appointment systems may lack sufficient flexibility for individuals requiring caregiver support or accessible transportation, digital platforms may not comply with accessibility standards, and prevention services may not routinely incorporate disability-sensitive indicators in their evaluation. These examples illustrate potential implementation barriers identified from the broader accessibility literature rather than barriers that have been systematically quantified across PrEP programmes.

These observations highlight a broader issue within implementation science. Biomedical interventions are often evaluated according to efficacy, safety and cost-effectiveness, whereas equitable delivery across populations with different accessibility needs may receive less explicit attention [22]. An intervention may demonstrate excellent efficacy under trial conditions while its real-world reach and effectiveness may be constrained if implementation does not account for communication needs, physical accessibility or functional diversity. Thus, pharmacological efficacy alone does not determine population-level impact; implementation context also matters.

Importantly, these barriers rarely occur in isolation. Individuals with disabilities may experience intersecting forms of disadvantage related to gender, socioeconomic position, migration status, ethnicity, sexual orientation or gender identity [8,21,41]. These intersections may shape both exposure to HIV-related vulnerabilities and the type or magnitude of barriers encountered across the prevention continuum. Available HIV prevention research rarely provides sufficiently disaggregated data to examine these intersections directly, representing an important evidence gap [22]. An intersectional perspective is therefore important for understanding why apparently universal prevention programmes may not produce equivalent access or outcomes across different populations.

Taken together, the literature suggests that inequities may emerge at multiple points across the HIV prevention continuum. However, much of the evidence currently supporting this framework comes from studies of sexual and reproductive healthcare, healthcare accessibility and disability more broadly, rather than from studies directly measuring PrEP uptake, persistence or outcomes among people with disabilities [3,12,13,14,15,16,17,18,20]. This distinction is important: the framework identifies plausible and evidence-informed implementation barriers, while also exposing the need for direct empirical evaluation within contemporary HIV prevention programmes. Biomedical innovation has transformed what HIV prevention can achieve, but its impact ultimately depends on whether increasingly sophisticated interventions remain accessible throughout every stage of delivery. This observation naturally raises the next question: has disability been adequately considered during the development and implementation of biomedical HIV prevention itself?

## 6. Biomedical HIV Prevention: The Missing Disability Dimension

Few areas of infectious diseases have advanced as rapidly as biomedical HIV prevention. Within little more than a decade, oral PrEP has become a cornerstone of HIV prevention, long-acting injectable cabotegravir has expanded preventive options for individuals unable or unwilling to take daily medication, and HIV self-testing has increased opportunities for early diagnosis and linkage to care [1,42,43,44]. Together, these innovations have fundamentally changed the prevention landscape, shifting attention from behavioural risk reduction alone towards integrated models combining pharmacological interventions, routine testing and personalised care.

Yet, despite this transformation, one question has received remarkably little attention: who is actually able to benefit from these advances?

Our review suggests that disability remains one of the least explored dimensions of biomedical HIV prevention. While hundreds of studies have examined PrEP awareness, uptake, adherence, persistence and implementation among populations such as gay, bisexual and other men who have sex with men, transgender people, sex workers, people who inject drugs and adolescents, people with disabilities are rarely identified as a distinct population within these investigations [22]. Direct empirical evidence specifically evaluating PrEP uptake, persistence, effectiveness or implementation according to disability status remains particularly limited. This omission is striking because disability is not a rare condition confined to a small subgroup of society. It affects more than one billion people globally and intersects with every demographic category routinely considered within HIV prevention programmes [2].

Importantly, the available evidence does not suggest deliberate exclusion. Rather, disability appears to have remained outside the conceptual framework through which biomedical prevention has evolved. Early PrEP programmes were understandably designed around populations with the highest documented HIV incidence, and subsequent implementation studies focused on expanding access within those same groups. Disability, by contrast, was seldom considered either as an independent equity dimension or as a factor capable of influencing implementation. Consequently, contemporary HIV prevention has become increasingly sophisticated while the accessibility of its delivery models for people with different types of disabilities remains poorly characterised [16,21].

This distinction is particularly important when considering pharmacological efficacy. The pivotal evidence supporting oral tenofovir-based PrEP and long-acting agents derives from trials conducted in populations selected primarily according to HIV exposure and acquisition risk rather than disability status. Disability-specific pharmacokinetic, pharmacodynamic and clinical endpoint data are sparse, and the available literature does not allow comparative estimates of preventive efficacy according to disability type. At the same time, there is no established evidence that disability as a broad category intrinsically modifies the pharmacological activity of PrEP. The principal evidence gap therefore concerns not demonstrated reduced efficacy, but the absence of disability-specific data and the limited understanding of how accessibility and service-delivery factors influence real-world use.

For this reason, disability should not be framed exclusively as either a pharmacological or an implementation issue. For many people with disabilities, the more immediate concerns are likely to arise along the pathway through which biomedical prevention is accessed and maintained. Awareness of PrEP, accurate assessment of HIV risk, timely HIV testing, access to prescribing clinicians, appropriate follow-up, laboratory monitoring and sustained engagement with care all represent components of effective prevention. The broader disability and sexual healthcare literature identifies barriers at several analogous stages, although their specific effects on PrEP uptake and persistence have not been adequately quantified [3,12,13,14,15,16,17,18,20]. Discriminatory attitudes and broader structural barriers within healthcare systems may further influence equitable access to biomedical prevention [4,8,15]. The absence of disability-specific PrEP studies should therefore be interpreted primarily as an evidence gap rather than as evidence either for or against the presence of implementation inequities.

The same considerations apply to newer biomedical prevention strategies. Long-acting injectable PrEP represents an important advance capable of overcoming some of the adherence challenges associated with daily oral medication [42,43,44]. From a disability perspective, this modality may offer meaningful advantages for some individuals. Removing the need for daily pill-taking may reduce medication-management burden, particularly for people who experience difficulties maintaining a daily regimen or who require support with medication routines. Long-acting administration may also offer greater discretion and reduce the day-to-day burden associated with oral prevention. These potential advantages are particularly relevant because accessibility is not limited to physical access to healthcare but also includes the ability to use and sustain a prevention strategy in everyday life.

At the same time, these advantages should not be assumed to apply uniformly across all people with disabilities. The balance between convenience and accessibility is likely to vary according to disability type, individual preference, support needs and healthcare context. For some people with intellectual or cognitive disabilities, reducing the need for daily pill-taking could potentially simplify adherence, provided that informed decision-making and appropriate support are ensured. Conversely, for some people with substantial mobility limitations, scheduled clinic attendance for injectable administration may introduce transportation or physical-access barriers. Sensory disabilities may raise different considerations, centred less on the route of administration than on accessible counselling, appointment systems and communication. Direct comparative studies evaluating these trade-offs among people with different disabilities are currently lacking. Long-acting formulations should therefore be understood as an important expansion of prevention choice that may provide substantial accessibility advantages for some people with disabilities, rather than as a universally preferable option.

Similar considerations apply to twice-yearly lenacapavir. Its infrequent dosing schedule may reduce some forms of treatment burden and the number of prevention-related dosing events, but disability-specific evidence on acceptability, accessibility, persistence and clinical outcomes is currently lacking. The practical implications of twice-yearly administration will therefore depend not only on dosing frequency but also on whether individuals can access appropriate clinical services and follow-up [45].

Similarly, HIV self-testing has emerged as a promising strategy for expanding HIV diagnosis by reducing dependence on facility-based testing and increasing autonomy. From a disability perspective, however, self-testing illustrates both the opportunities and limitations of biomedical innovation [1,19].

Accessible self-testing could reduce transportation barriers, increase privacy and facilitate earlier diagnosis. At the same time, the extent to which HIV self-testing approaches have been evaluated for accessibility across different disability groups remains poorly characterised. People with sensory, physical, intellectual or cognitive disabilities may encounter different accessibility requirements related to test instructions, manipulation of test components, result interpretation or access to appropriate support. These considerations represent plausible accessibility requirements, but direct HIV self-testing studies examining them remain limited. Accessibility cannot therefore be viewed as an optional refinement introduced after technological development; it should be considered during the design, evaluation and implementation of self-testing programmes.

Another important observation emerging from the literature concerns the way equity is conceptualised within HIV prevention. Contemporary implementation frameworks increasingly recognise the importance of reducing disparities associated with gender, ethnicity, socioeconomic disadvantage and sexual orientation. Disability, however, is seldom included within these discussions despite representing a major dimension of healthcare accessibility and health equity worldwide [2,22]. This omission is particularly relevant because disability frequently coexists with many of these other determinants of vulnerability. A woman with a physical disability, a Deaf migrant, or an individual with intellectual disability who identifies as a sexual minority may experience multiple, interacting barriers that are unlikely to be addressed by implementation strategies focusing on only a single dimension of inequity. However, intersectional analyses incorporating both disability and these other determinants remain scarce within the biomedical HIV prevention literature.

Ultimately, the literature points towards a broader conceptual issue. Biomedical HIV prevention has largely been evaluated according to whether interventions work, whereas considerably less attention has been devoted to whether healthcare systems are capable of delivering those interventions equitably. Disability exposes this gap particularly clearly. It also demonstrates why accessibility cannot be inferred from dosing convenience alone: oral, injectable and self-administered prevention strategies may each remove some barriers while introducing or retaining others. The most appropriate option is therefore likely to depend on individual accessibility needs, preferences and care context rather than disability status alone.

For this reason, disability should not be regarded as another niche subgroup requiring separate prevention programmes. Instead, it should be recognised as a lens through which the inclusiveness of contemporary HIV prevention can be critically evaluated. The current priority is therefore twofold: to generate disability-disaggregated evidence on access, uptake, persistence and outcomes, and to incorporate accessibility prospectively into the design and evaluation of prevention pathways [21]. In this way, accessibility can become an integral component of biomedical HIV prevention rather than an afterthought.

## 7. Toward Disability-Inclusive HIV Prevention: From Accessibility to Implementation

Recognising disability as an overlooked dimension of HIV prevention is only the first step. The greater challenge lies in translating this recognition into prevention programmes capable of delivering biomedical innovations equitably. Achieving this goal will require more than adapting existing services. It calls for a shift in perspective, from viewing disability as a characteristic of individuals to recognising accessibility as a defining feature of high-quality HIV prevention systems.

This distinction is crucial. Historically, disability has often been addressed through specialised services operating alongside mainstream healthcare. Although these programmes remain essential for many individuals, they are unlikely to eliminate disparities in HIV prevention if routine sexual health services continue to be designed around the needs of people without disabilities. Biomedical prevention has become increasingly integrated into primary care, sexual health clinics and community-based services. Consequently, accessibility should be considered an important component of equitable HIV programme implementation rather than solely an additional accommodation introduced after services have been designed [2,4].

The first priority is to consider accessibility during the design of prevention services. Recommendations derived from disability-inclusive healthcare frameworks include attention to physical accessibility. HIV testing centres, sexual health clinics and PrEP services may need to address barrier-free access, accessible examination facilities and transportation barriers, particularly for people with mobility limitations [3,12,13,15,16,32]. Yet accessibility extends well beyond architecture. Communication is equally important. Prevention programmes may improve accessibility by providing information and communication in formats adapted to different sensory, intellectual or cognitive accessibility needs [17,18,19,31,36,38,40].

These adaptations represent practical approaches proposed within disability-inclusive and person-centred healthcare, although their specific effects on HIV prevention uptake and outcomes have not been adequately evaluated [12,13,14,15,16,17,18].

Healthcare professionals represent another key component of disability-inclusive prevention. Much of the literature reviewed in this manuscript suggests that provider assumptions may contribute to exclusion but should be considered alongside structural, organisational and communication barriers. Sexual histories may not be explored when disability is assumed to imply sexual inactivity. HIV prevention may not be discussed when exposure or prevention needs are underestimated, and PrEP may never be offered if eligibility is not considered. Addressing these issues may involve professional training in communication, supported and shared decision-making and inclusive sexual healthcare [15,16,17,18,36,38]. However, evidence demonstrating that provider training alone improves HIV-specific prevention outcomes among people with disabilities remains limited. Training should therefore be considered one component of a broader accessibility strategy rather than a stand-alone solution. The principal structural barriers identified in the literature, together with their potential impact on the HIV prevention continuum and corresponding evidence-informed disability-inclusive implementation strategies, are summarized in Table 1.

Implementation research should also evolve. Despite substantial progress in PrEP delivery science, disability remains poorly characterised within much of the implementation literature reviewed here. Future studies should routinely record disability status, evaluate disability-disaggregated outcomes and explore barriers affecting awareness, uptake, adherence and persistence in prevention programmes [21]. Potential implementation outcomes include PrEP awareness, PrEP offer and initiation, persistence or retention, completed and missed visits, successful access to HIV self-testing, and patient-reported measures of accessibility and acceptability (Table 1).

Importantly, disability should not simply be added as another demographic variable within statistical models. Instead, where feasible, disability type and relevant functional or accessibility needs should also be considered, because aggregate disability measures may obscure important differences between people with mobility, sensory, intellectual or cognitive disabilities. An intersectional approach should additionally examine how disability interacts with gender, sexual orientation, gender identity, migration status and socioeconomic position. Incorporating these dimensions into implementation frameworks from the outset could help investigators identify where along the prevention continuum inequities emerge and evaluate which approaches are most appropriate for different populations and contexts.

This represents an opportunity rather than merely a research gap. Contemporary implementation science increasingly emphasises context, adaptability and person-centred delivery. Disability offers a particularly valuable lens through which these concepts can be examined because it may reveal accessibility weaknesses that are not apparent when prevention systems are evaluated without considering functional diversity. In this sense, disability-inclusive HIV prevention has implications extending well beyond disability itself. Improving accessibility may also benefit other individuals facing communication barriers, reduced health literacy, frailty or complex healthcare needs [12,13,14,15].

The implementation of disability-inclusive biomedical HIV prevention should also be understood as context dependent. Prevention pathways cannot be assumed to operate similarly across countries, as access to PrEP modalities, healthcare financing, service organisation, transportation, digital infrastructure and disability-related support may differ substantially between settings. Strategies that improve accessibility in one health system may therefore be less feasible or require adaptation in another. Rather than proposing a single global model, disability-inclusive HIV prevention should combine common principles of equity and accessibility with implementation approaches adapted to national and local healthcare contexts.

Policy development must evolve in parallel. Although disability is increasingly recognised within international public health agendas, it remains variably addressed in the HIV-related policy and guideline documents examined in this review [1,3,5,21,32,42,43,44,45,46,47].

Our comparison of major contemporary HIV prevention guidelines indicates uneven consideration of disability and accessibility (Table 2). WHO guidance explicitly recognises accessibility within broader person-centred HIV service delivery, while BASHH/BHIVA provides comparatively detailed recommendations addressing PrEP equity and physical, digital and organisational barriers to access. However, across the guidelines examined, disability is rarely operationalised as a distinct equity dimension: specific disability types are generally not differentiated, disability-specific accommodations are seldom described, and disability-disaggregated implementation or monitoring indicators are largely absent [1,42,43,44,45,46,47].

Future policy documents could move beyond acknowledging disability as a vulnerable condition and instead define measurable objectives related to accessibility, service utilisation and equitable prevention coverage. Monitoring disability-disaggregated indicators could provide a means of assessing whether biomedical prevention is reaching people with different accessibility needs.

The rapid expansion of digital health presents additional opportunities. Telemedicine, electronic appointment systems, remote adherence support and mobile health applications are becoming integral components of HIV prevention. When developed according to accessibility and universal design principles, these technologies may reduce some barriers for some people with disabilities; however, the benefit is unlikely to be uniform across disability types or settings [31,48]. Emerging digital approaches may further expand these possibilities. Digitally delivered educational interventions, potentially including appropriately designed AI-supported tools, could offer more adaptable ways of providing sexual health and HIV prevention information, although their accessibility and effectiveness for people with different disabilities require specific evaluation [48]. Innovative delivery models may also help address geographical barriers to conventional healthcare delivery. Drone-supported delivery of HIV medication has already been explored in hard-to-reach communities, suggesting a potential role for such technologies where conventional access is difficult [49]. Whether these approaches can specifically improve access to HIV prevention, testing or treatment for people with disabilities remains to be established. Conversely, if accessibility is overlooked during development, digital innovation may introduce or reinforce inequities. Accessibility should therefore be considered during design and evaluation rather than only as a later corrective measure.

Ultimately, disability-inclusive HIV prevention is not simply about expanding access to PrEP. It is about rethinking how prevention is delivered. The evolution of HIV prevention over the past decade has demonstrated that scientific innovation alone is insufficient. Biomedical efficacy can only translate into population-level impact when healthcare systems are capable of delivering interventions consistently, equitably and in ways that accommodate the diversity of those they serve. At present, many of the strategies proposed to improve disability inclusion are supported more strongly by broader accessibility and disability-health evidence than by direct HIV-specific intervention studies. Their inclusion here should therefore be understood as an implementation agenda requiring prospective evaluation rather than as a set of interventions with already established HIV-specific effectiveness.

## 8. Conclusions

The history of HIV prevention is one of continuous innovation. Behavioural interventions transformed the early response to the epidemic, antiretroviral therapy altered the natural history of HIV infection, and biomedical prevention has redefined what is now achievable before infection occurs. Oral PrEP, long-acting injectable prophylaxis and HIV self-testing have collectively moved HIV prevention into an era in which eliminating new infections is increasingly viewed as a realistic public health objective.

Our review suggests, however, that this scientific progress has not been matched by equivalent progress in accessibility. A substantial body of evidence demonstrates that people with disabilities continue to experience barriers across sexual and reproductive healthcare, HIV education, testing and preventive services. In contrast, disability remains poorly represented in the rapidly expanding literature on biomedical HIV prevention. Importantly, the evidence is considerably stronger for inequalities in broader sexual and reproductive healthcare and healthcare accessibility than for disability-specific differences in PrEP uptake, persistence or outcomes. This imbalance represents an important evidence gap and indicates that disability has received limited attention as an accessibility and equity dimension within biomedical HIV prevention research and implementation.

The central message emerging from the available literature is therefore not that disability determines either HIV risk or the effectiveness of biomedical prevention, but that disability-related accessibility needs may influence whether individuals can benefit from available prevention options. Disability-specific pharmacokinetic, pharmacodynamic and clinical endpoint data remain sparse, while direct implementation evidence is also limited. The challenge is therefore to generate better disability-specific evidence while ensuring that prevention systems can respond to heterogeneous accessibility needs. Accessibility, communication, health literacy, service design and provider attitudes may influence different stages of the prevention continuum, although their effects are likely to vary according to disability type, individual circumstances and healthcare context.

Viewing disability through this lens has implications extending beyond HIV medicine. It shifts the discussion from identifying vulnerable populations to evaluating the inclusiveness of healthcare systems themselves. A prevention programme capable of meeting the needs of people with disabilities may also be more responsive to other populations experiencing structural barriers to care. Disability therefore becomes not only an equity issue but also a useful dimension through which healthcare accessibility and quality can be evaluated.

As HIV prevention continues to evolve, future research should move beyond documenting disparities and begin evaluating disability-inclusive implementation strategies. Clinical trials, implementation studies and national prevention programmes should consider incorporating disability as an equity dimension, generate disability-disaggregated data and prospectively evaluate accessibility across different disability types and prevention modalities. Research should also determine whether oral, long-acting and self-administered prevention options create different accessibility advantages or barriers for people with mobility, sensory, intellectual or cognitive disabilities, including when disability intersects with gender, sexual orientation, gender identity, migration status and socioeconomic position. The accessibility strategies proposed in this review should be prospectively evaluated for their effects on HIV-specific outcomes rather than assumed to be effective on the basis of broader disability-health evidence alone. Integrating accessibility into biomedical HIV prevention from the outset may help ensure that scientific advances translate into more equitable population-level benefit.

## Figures and Tables

**Table 1:** Structural barriers, impact across the HIV prevention continuum, and potential strategies for disability-inclusive biomedical HIV prevention.

Structural Barrier	Potential Impact Across the HIV Prevention Continuum	Potential Disability-Inclusive Strategies	Potential Implementation Outcomes
Physical inaccessibility	Reduced access to HIV testing, PrEP services, follow-up visits and long-acting injectable PrEP [3,12,13,15]	Barrier-free clinical environments, accessible examination facilities and attention to transportation barriers	PrEP initiation; completed and missed visits; retention in prevention services
Communication barriers	Reduced understanding of HIV risk, PrEP counselling and informed decision-making [12,17,18,38,40]	Accessible information and communication formats adapted to sensory, intellectual and cognitive accessibility needs	Successful completion of PrEP counselling; patient-reported communication accessibility; informed participation in prevention decisions
Limited sexual health education and health literacy	Lower awareness of HIV prevention strategies, delayed healthcare seeking and potential barriers to PrEP uptake [11,28,29,30,39]	Inclusive sexuality education, accessible HIV prevention campaigns and disability-adapted educational resources	PrEP awareness; knowledge of available prevention options; successful access to HIV self-testing
Healthcare provider bias and insufficient training	Missed opportunities for sexual health assessment, HIV testing and PrEP prescription [15,17,18,40]	Training in disability-inclusive sexual healthcare, routine sexual history taking and shared decision-making	PrEP offer; HIV testing offer; PrEP initiation; patient-reported experience of care
Social vulnerability and exposure to sexual violence	Potentially increased exposure to HIV-related vulnerability and barriers to engagement with prevention services [9,26,27]	Trauma-informed care, integrated psychosocial support and safeguarding programmes	Engagement with prevention services; retention in care; patient-reported accessibility and acceptability
Policy and implementation gaps	Limited integration of disability into HIV prevention programmes and monitoring systems [3,4,5,21,22,32]	Disability-inclusive HIV policies, implementation research, disability-disaggregated indicators and equitable service evaluation	Disability-disaggregated PrEP awareness, offer, initiation and persistence; equitable prevention coverage

People with disabilities may encounter multiple structural barriers that may influence different stages of the HIV prevention continuum, from awareness and HIV testing to PrEP initiation, adherence and long-term retention in care. The proposed strategies are evidence-informed approaches derived from the broader disability, accessibility and sexual healthcare literature; their effectiveness in improving HIV-specific prevention outcomes among people with disabilities requires direct evaluation.

**Table 2:** Comparative consideration of disability and accessibility in major contemporary HIV prevention guidelines.

Guideline	Explicit Mention of Disability	General Equity/Accessibility Framework	Specific Disability Types Differentiated	Practical Disability-Specific Accommodations	Disability-Specific Implementation or Monitoring
WHO Consolidated HIV Guidelines, 2021 [1]	Yes	Yes	Limited	Limited/partial	Limited
WHO Lenacapavir PrEP Guideline, 2025 [45]	No clear disability-specific focus identified	Yes	No	No specific accommodations identified	No disability-specific monitoring identified
CDC Clinical Guidance for PrEP, 2026 [42]	No explicit mention identified	Partial	No	No specific accommodations identified	No
BASHH/BHIVA PrEP Guidelines, 2025 [43]	No explicit mention identified	Yes—strong	No	No disability-specific accommodations identified	No disability-specific monitoring identified
IAS–USA Recommendations, 2024/2025 [44]	No explicit mention identified	Limited/implicit	No	No specific accommodations identified	No
WHO Consolidated HIV Guidelines: Service Delivery, 2026 [46]	No clear PrEP-specific disability framework identified	Yes—strong	No clear differentiation identified	No PrEP-specific disability accommodations identified	No disability-specific PrEP monitoring identified
EACS Guidelines v13.0, 2025 [47]	No explicit disability mention identified	Limited in PrEP guidance	No in PrEP context	No specific accommodations identified	No

Major contemporary HIV prevention guidelines were compared for the extent to which disability and accessibility are explicitly incorporated into recommendations, including recognition of disability, general equity and accessibility principles, differentiation by disability type, practical accommodations, and disability-specific implementation or monitoring. Although accessibility and equity are addressed to varying degrees, particularly in WHO and BASHH/BHIVA guidance, disability is rarely operationalised as a distinct dimension of biomedical HIV prevention. Abbreviations: BASHH, British Association for Sexual Health and HIV; BHIVA, British HIV Association; CDC, Centers for Disease Control and Prevention; EACS, European AIDS Clinical Society; HIV, human immunodeficiency virus; IAS–USA, International Antiviral Society–USA; PrEP, pre-exposure prophylaxis; WHO, World Health Organization. Note: Guidelines were assessed using the predefined terms disability, disabled, accessibility, impairment, wheelchair, cognitive, intellectual, deaf, hearing, blind, visual, and reasonable accommodation. References to clinical impairment (e.g., renal impairment) were not considered evidence of disability inclusion. “Partial” or “limited” indicates that equity or accessibility was addressed broadly without comprehensive operationalisation for people with disabilities.

## Data Availability

No new data were created or analyzed in this study. Data sharing is not applicable to this article.

## Abbreviations

HIV	Human immunodeficiency virus
PrEP	pre-exposure prophylaxis
STI	sexually transmitted infections
WHO	World Health Organization
CDC	Centers for Disease Control and Prevention
EACS	European AIDS Clinical Society
BHIVA	British HIV Association
IAS–USA	International Antiviral Society–USA
